# Transmission of COVID-19 among healthcare workers-an epidemiological study during the first phase of the pandemic in Sweden

**DOI:** 10.1017/S0950268822000231

**Published:** 2022-03-11

**Authors:** Sekai Chenai Mathabire Rücker, Catharina Gustavsson, Fredrik Rücker, Anders Lindblom, Maria Hårdstedt

**Affiliations:** 1 Department of Infectious Diseases, Falun Hospital, Falu lasarett, SE-79182 Falun, Sweden; 2 Center for Clinical Research Dalarna – Uppsala University, Nissers väg 3, SE-79182 Falun, Sweden; 3 School of Health and Welfare, Dalarna University, SE-79188 Falun, Sweden; 4 Department of Public Health and Caring Sciences, Uppsala University, BMC, Box 564, SE-751 22 Uppsala, Sweden; 5 Unit of Infectious Diseases, Department of Medical Sciences, Uppsala University, Akademiska sjukhuset, SE-751 85 Uppsala, Sweden; 6 Department of Infection Control Dalarna, Falun Hospital, Falu lasarett, SE-79182 Falun, Sweden; 7 School of Medical Sciences, Faculty of Medicine and Health, Örebro University, SE-70182 Örebro, Sweden; 8 Vansbro Primary Health Care Center, Moravägen 27, SE-78633 Vansbro, Sweden

**Keywords:** COVID-19, health care workers, infection prevention and control, personal protective equipment, Sweden

## Abstract

During the first phase of the COVID-19 pandemic in 2020, concerns were raised that healthcare workers (HCWs) were at high risk of infection. The aim of this study was to explore the transmission of COVID-19 among HCWs during a staff outbreak at an inpatient ward in Sweden 1 March to 31 May 2020. A mixed-methods approach was applied using several data sources. In total, 152 of 176 HCWs participated. The incidence of COVID-19 among HCWs was 33%. Among cases, 48 (96%) performed activities involving direct contact with COVID-19 patients. Contact tracing connected 78% of cases to interaction with another contagious co-worker. Only a few HCW cases reported contact with a confirmed COVID-19 case at home (*n* = 6; 12%) or in the community (*n* = 3; 6%). Multiple logistic regression identified *direct care of COVID-19 patients* and *positive COVID-19 family contact* as risk factors for infection (adjusted OR 8.4 and 9.0 respectively). Main interventions to stop the outbreak were physical distancing between HCWs, reinforcement of personal hygiene routines and rigorous surface cleaning. The personal protective equipment used in contact with patients was not changed in response to the outbreak. We highlight HCW-to-HCW transmission of COVID-19 in a hospital environment and the importance of preventing droplet and contact transmission between co-workers.

## Introduction

Coronavirus disease 2019 (COVID-19), caused by severe acute respiratory syndrome coronavirus 2 (SARS-CoV-2), was declared a global pandemic by the World Health Organization (WHO) on 11 March 2020 [1]. The first case of COVID-19 was reported in Sweden on 31 January and on 10 March 2020 the Swedish Public Health Agency officially declared a societal spread of COVID-19 in Sweden [2]. The first phase of the pandemic lasted until the end of July 2020. During this first phase, high numbers of infections and deaths due to COVID-19 among healthcare workers (HCWs) raised concerns globally [3, 4]. Lack of knowledge on transmission routes of SARS-CoV-2 in a hospital environment and shortage of adequate personal protective equipment (PPE) added to insecurity and stress among HCWs [5, 6]. Being a critical resource for the society during a pandemic, HCWs are crucial to protect [7–11]. HCWs are also a source of transmission of infection to other patients and to the overall community [12–14].

Nosocomial outbreaks of COVID-19 among HCWs have been reported together with plausible risk factors for COVID-19 infections among HCWs [15–21]. However, there is a shortage of studies mapping workplace mechanisms behind local outbreaks and measures taken for infection control. Due to multifactorial mechanisms, studying virus transmission and infection control in a real-life workplace environment is challenging. Attempts to systematically explore such mechanisms are crucial for timely interventions to secure the health of HCWs and prevent HCWs from being a source of infection during future infectious outbreaks.

The aim of this study was to explore means of transmission and measures of control during an outbreak of COVID-19 among HCWs at an inpatient ward of Infectious Diseases in Sweden. By using a mixed-method approach, we aimed to map individual and organisational factors contributing to infection transmission and control.

## Methods

### Study design

A retrospective epidemiological observational study was performed for the period 1 March to 31 May 2020. Mixed methods with a convergent approach were used for data collection and analysis [22].

### Study population and setting

The study was based on an outbreak of COVID-19 among HCWs at an inpatient ward of Infectious Diseases at a county hospital in one healthcare region in Sweden. All staff working at the ward during the study period were eligible; i.e. physicians, nurses, nurse aides, physiotherapists, rehabilitation assistants, kitchen assistants, cleaning staff and medical secretaries. In addition, three persons in management positions were selected for interviews. Ethical approval was obtained from the Swedish Ethical Review Authority (Dnr 2020-03120).

The ward consists of 22 isolation rooms with sluice rooms, two rooms with double beds and surrounding working areas (Supplementary Fig. S1; Supplementary material available on the Cambridge Core website). During the study period, the hospital supplied care for all hospitalised patients with COVID-19 within a catchment population of 270 000. Oxygen treatment was delivered by nasal cannula or face mask and high flow nasal cannula oxygen therapy was implemented from 19 March 2020. Patients in need of invasive mechanic ventilation were transferred to the intensive care unit within the hospital.

The infection prevention and control (IPC) measures implemented at the ward during the COVID-19 pandemic were based on recommendations from the Public Health Agency of Sweden and the National Board of Health and Welfare. Testing for SARS-CoV-2 RNA by reverse transcription real-time polymerase chain reaction in Sweden started in early March 2020. At this county hospital, polymerase chain reaction (PCR) analysis started on 23 March 2020. During the study period, testing capacity was very limited. Only generally affected patients with suspected respiratory infections admitted to the hospital or seeking help at the emergency department were routinely tested for SARS-CoV2. Testing of HCWs with symptoms was started mid-March, however selective for personnel needed in COVID-19 patient care. Patients admitted to the hospital were tested if developing respiratory symptoms and/orfever.

### Data collection

Data collection was performed from July to August 2020 by several data sources:

- A self-administered questionnaire with closed and open-ended questions to all HCWs on COVID-19 exposure, symptoms and results of testing. Self-reported knowledge of PPE in four hypothetical activities involving patientcare was also examined (Supplementary Fig. S2).
- Daily work shift records for information on working days and hours, as well as work area. Relevant contacts between HCWs were considered as (A) worked the same shift caring for different group of patients, (B) worked the same shift caring for the same group of patients and (C) cared for the same group of patients in consecutive shifts (meeting for handover reports) (Supplementary Table S1).
- The hospital's database for daily numbers of COVID-19 patients at the ward.
- Individual interviews with three persons in management position on their views on IPC measures, means of transmission during the staff outbreak and strategies to stop the outbreak. The interviews were recorded and transcribed into text.
- Notes from daily and weekly workplace meetings were assessed for information from the management to the HCWs relating IPC measures.
- Laboratory data: SARS-CoV-2 RNA was analysed on a nasopharyngeal and oropharyngeal swab according to Corman *et al.* with modifications [23]. IgG (N) was analysed by the Abbott Architect™ SARS-CoV-2 IgG test (Abbott Diagnostics, IL, USA).

### Case definitions

*Confirmed COVID-19 case*: person with symptoms testing positive for SARS-CoV-2 RNA [24]. SARS-CoV-2 PCR tests up to 14 June 2020 were considered based on a 14-day incubation period [25]. *Suspected COVID-19 case*: person meeting the clinical criteria (with at least one of the following symptoms: cough, fever, shortness of breath, sudden onset of anosmia, ageusia or dysgeusia) in combination with an epidemiological link (close contact with a confirmed COVID-19 case during 14 days prior to onset of symptoms or presence of antibodies to SARS-CoV-2 during the study period) [24]. COVID-19 cases were considered infectious 48 h before symptom onset and 10–14 days after, depending on symptom severity [26].

### Data analysis

Questionnaire data were analysed by descriptive statistics. Responses to open-ended questions were categorised and summarised in numbers and proportions. Contact tracing and mapping of transmission was performed manually based on questionnaires and daily work records. Simple and multiple logistic regression analyses were performed to assess factors associated with COVID-19 infection and reported as unadjusted (OR) and adjusted (aOR) odds ratios. Explanatory variables for multiple regression analysis were selected based on association with COVID-19 infection in simple regression analysis (*positive family contact*; *working in direct care of patients with COVID-19*; *correct knowledge of PPE*) and were adjusted for age and sex [27]. All reported symptoms were included in the multiple regression model. Knowledge of PPE routines was quantified accordingly: 100% referred to correct intended use of PPE for all four clinical scenarios, 75% referred to one scenario incorrect, 50% two scenarios incorrect and <50% to more than two scenarios reported incorrectly. Stata software version 16 (College Station, Texas, USA) was used for statistical analysis.

Qualitative content analysis with an inductive approach was applied for analysis of interviews with the managers [28, 29]. Interview transcripts were analysed in Atlas TI software 9 (Scientific Software Development GmbH, Berlin Germany). Units of data were coded, and grouped in subcategories and main categories.

## Results

Of 176 people working at the ward between 1 March and 31 May 2020, 152 participated in the study. Seventeen did not respond and seven declined participation. A majority of the participants worked in direct contact with patients (*n* = 125; 82%) (Supplementary Table S2). Approximately half (*n* = 69; 45%) were from other clinics and worked temporarily at the ward.

### Outbreak of COVID-19 among HCWs

The first case of PCR-verified COVID-19 was admitted to the ward on 12 March 2020. During the study period, 970 days of care were reported for patients with COVID-19 and 721 days of care for patients with other infectious diseases. No patient admitted for another infection developed symptoms of COVID-19, followed by a positive test for SARS-CoV-2.

**Fig. 1.**
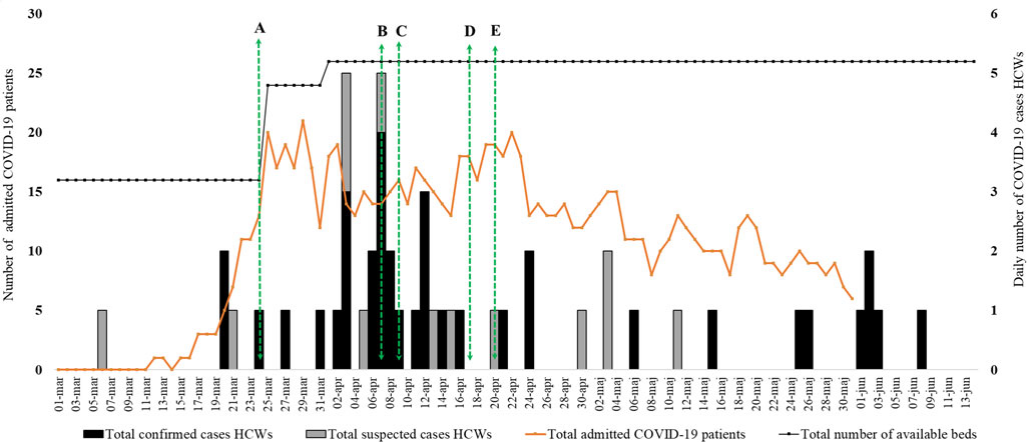
Epidemic curve of the COVID-19 outbreak among HCWs at the infectious disease ward. Confirmed (black bars) and suspected cases (grey bars) of COVID-19 among HCWs at the ward. Total daily number of admitted patients with COVID-19 at the care unit is presented as an orange line, total bed occupancy is presented as a black line. Green arrows present time points for implementation of selected IPC measures: (A) increased frequency of disinfection of inanimate objects at the ward and reinforcement of personal hygiene practices; (B) ‘Friday tea’ cancelled from this day; (C) daily staff meetings took place in two groups instead of one from this day; (D) shared face shields were changed twice daily from this date; (E) increased frequencies of cleaning of shared areas such as lunch room and staff toilets. HCWs, health care workers; IPC, infection prevention and control.

In the beginning of April, an outbreak of COVID-19 was apparent among HCWs at the ward (Fig. 1). During the study period, there were 36 (24%) confirmed cases and 14 (9%) suspected cases of COVID-19 among the HCWs, corresponding to an incidence of 33% (Table 1). Of 102 HCWs not defined as COVID-19 cases, 72 (71%) reported one or more symptoms related to COVID-19 and of these, 40 (56%) were never tested for SARS-CoV-2. The presence of anosmia and/or ageusia was associated with COVID-19 infection (aOR 9.9; 95% CI 3.3–29.4; *P* < 0.001), whilst those with abdominal symptoms were less likely to have COVID-19 (aOR 0.2; 95% CI 0.05–0.7; *P* = 0.011) (Table 2). The majority of HCWs with COVID-19 experienced mild to moderate symptoms, and only one person needed hospital care.

**Table 1.** Background characteristics, reported symptoms and possible exposures to COVID-19 of HCWs stratified by SARS-CoV-2 test status

Characteristics/exposures	Confirmed COVID-19 cases (*n* = 36)	Suspected COVID-19 cases (*n* = 14)	Non-cases (never tested or negative) (*n* = 102)
Background characteristics			
Female, *n* (%)	30 (83)	14 (100)	86 (84)
Age: median (IQR)	33 (27–47)	29 (26–45)	35 (26–47)
Reported an underlying condition defined as risk factor for severe COVID-19 infection^a^, *n* (%)	12 (33)	2 (14)	33 (33)
Years of work experience: median (IQR)	6.5 (2–20)	7 (4–11)	6 (2–16)
Works regularly at the ward; *n* (%)	19 (53)	10 (71)	46 (45)
Profession			
Physician, *n* (%)	5 (14)	1 (7)	16 (16)
Nurse, *n* (%)	17 (47)	7 (50)	33 (32)
Nurse aides, *n* (%)	9 (25)	5 (36)	27 (27)
Physiotherapist/rehabilitation assistants, *n* (%)	3 (8)	1 (7)	1 (1)
Cleaners, *n* (%)	2 (6)	0	19 (19)
Medical secretaries, *n* (%)	0	0	4 (4)
Kitchen assistants, *n* (%)	0	0	2 (2)
Possible exposure to COVID-19			
Reported history of recent travel abroad; *n* (%)	3 (9)	1 (7)	10 (10)
Reported history of a contact recently travelled abroad; *n* (%)	9 (25)	3 (21)	27 (26)
Positive COVID-19 contact outside home or work; *n* (%)	1 (3)	2 (14)	16 (16)
Positive COVID-19 family contact; *n* (%)	5 (14)	1 (7)	2 (2)
Regularly uses personal room; *n* (%)	22 (61)	12 (86)	59 (58)
Level of patient exposure			
Working in direct care of patients with COVID-19	34 (94)	14 (100)	73 (72)
Longer patient exposure (nurses + nurse assistants)	26 (72)	11 (79)	60 (59)
Shorter patient exposure (doctors + physiotherapists)	8 (22)	2 (14)	17 (17)
No patient related activities (cleaners, secretaries, kitchen assistants)	2 (6)	1 (7)	25 (25)
COVID-19 symptoms			
Any symptoms COVID-19, *n* (%)	36 (100)	12 (86)	72 (71)
Cough, *n* (%)	16 (44)	8 (67)	30 (42)
Fever, *n* (%)	20 (56)	7 (58)	24 (33)
Dyspnoea, *n* (%)	15 (42)	1 (8)	13 (18)
Runny nose, *n* (%)	21 (58)	9 (75)	41 (57)
Sore throat, *n* (%)	15 (42)	8 (67)	48 (67)
Headache, *n* (%)	22 (61)	10 (83)	54 (75)
Abdominal symptoms, *n* (%)	6 (17)	1 (8)	24 (33)
Muscle and joint aches, *n* (%)	25 (69)	3 (25)	20 (28)
Anosmia, *n* (%)	28 (78)	3 (25)	5 (7)
Ageusia, *n* (%)	27 (75)	3 (25)	9 (13)
Duration of symptoms; median (IQR)	12 (7–16)	5 (2–8)	

a The underlying conditions/risk factors were smoking, chronic lung disease, chronic heart and circulatory disease, diabetes.

**Table 2.** Reported symptoms and risk factors associated with COVID-19 among HCWs: simple and multiple logistic regression

	Simple logistic regression OR (95% CI)	Multiple logistic regression aOR (95% CI)
*Reported symptoms*		
Cough	1.4 (0.7–2.9)	0.7 (0.2–2.1)
Fever	**2.6** (**1.2–5.4)**^a^	2.6 (0.9–7.6)
Dyspnoea	2.3 (1.0–5.3)	1.5 (0.5–5.1)
Runny nose	1.3 (0.6–2.7)	1.6 (0.6–4.5)
Sore throat	**0.5** (**0.2–0.9)**^a^	0.6 (0.2–1.7)
Headache	0.7 (0.3–1.5)	0.4 (0.1–1.3)
Abdominal symptoms	**0.3** (**0.1–0.9)**^a^	**0.2 (0.04–0.7)**^a^
Muscle and joint aches	**3.6** (**1.6–7.7)**^a^	2.7 (0.9–8.6)
Anosmia or ageusia	**15.0** (**6.3–35.7)**^a^	**(3.3–29.4)**^a^
*Risk factors*		
Sex		
Female	ref	ref
Male	0.7 (0.3–2.0)	0.8 (0.2–2.5)
Age (years)	1.0 (1.0–1.0)	1.0 (0.9–1.0)
Reported an underlying risk condition (yes/no)	0.8 (0.4–1.7)	–
Work experience (years)		
<1 year	ref	–
1–5 years	2.0 (0.7–6.6)	–
>5 years	1.7 (0.6–4.6)	–
Works regularly at the ward (yes/no)	1.5 (0.8–3.1)	–
Reported history of recent travel abroad (yes/no)	0.8 (0.2–2.8)	–
Positive COVID-19 contact outside home/work (yes/no)	0.6 (0.3–1.2)	–
Positive COVID-19 family contact (yes/no)	**6.8** (**1.3–35.1)**^a^	**9.0** (**1.5–53.5)**^a^
Working in direct care of patients with COVID-19 (yes/no)	**9.2** (**2.1–40.4)**^a^	**8.4 (1.6–45.5)**^a^
Reported correct knowledge of PPE use (yes/no)	**2.5** (**1.2–5.2)**^a^	1.3 (0.5–3.1)

OR, odds ratio; aOR, adjusted odds ratio.

–, Variables not included in the multivariable analysis model.

a Figures in bold reflect statistical significant OR with significance level <0.05.

### Risk factors and possible routes of transmission

Among HCWs infected by SARS-CoV-2, 48 (96%) performed activities involving direct contact with COVID-19 patients. Only six (12%) and three (6%) HCWs reported contact with a confirmed COVID-19 case at home or in the community, respectively (Table 1). Simple logistic regression analysis (Table 2) showed that risk factors associated with acquiring COVID-19 infection in the adjusted logistics regression model were a *positive family contact* (aOR 9.0; 95% CI 1.5–53.5; *P* = 0.015), and *working in direct care of patients with COVID-1*9** (aOR 8.4; 95% CI 1.6–45.5; *P* = 0.013).

**Fig. 2.**
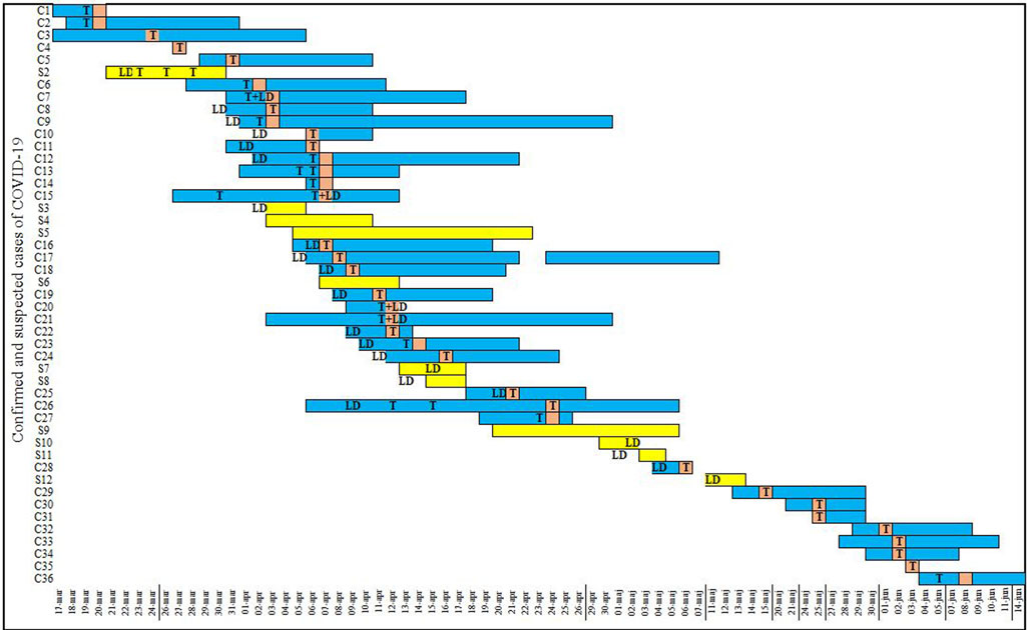
Duration of symptoms for *confirmed* and *suspected COVID-19 cases* among HCW. Confirmed cases (C1–C36) are presented with blue bars and suspected cases (S3–S12) with yellow bars. The date marked in orange represents the date when tested positive for SARS-CoV-2 PCR. The letter **T** represents the date of a PCR test; while **LD** denotes the last date worked at the infectious disease ward; and **T + LD** shows that the persons worked on the same day they tested positive. The figure presents data for all HCWs for which we have data on duration of symptoms (47 of 50). Two suspected cases did not experience symptoms of COVID-19 whilst one suspected case had missing data; thus they were excluded in this illustration. The *x*-axis presents dates and the vertical lines between some dates indicate dates excluded for a more compact layout.

**Fig. 3.**
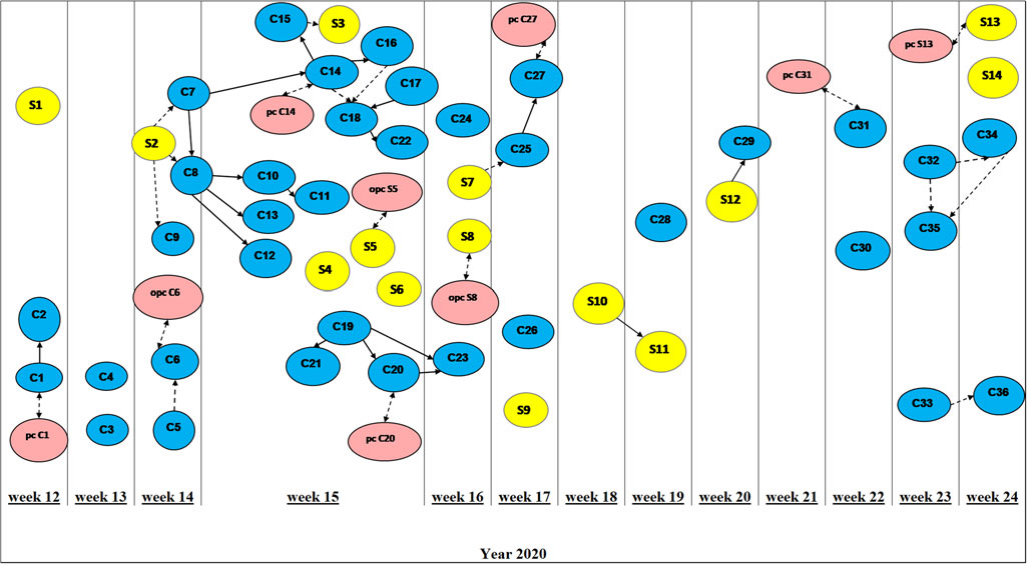
COVID-19 transmission tree based on contact tracing. Arrows pointing in the direction of assumed transmission according to the date of onset of symptoms. *Confirmed COVID-19 cases* are presented as *C1–C36* (blue bubbles). The numbers 1–36 indicate the order in which the cases tested positive for SARS-CoV-2. *Suspected COVID-19 cases* are presented as *S1–S14* (yellow bubbles). The numbers 1–14 indicate the order in which these suspected cases developed symptoms of COVID-19 or were identified as suspect cases based on contact tracing. The label ‘pc C1’ refers to a positive family contact of participant C1; ‘opcC6’ refers to a positive contact of C6 outside home and work. Only contacts with a confirmed infection based on PCR testing were included. *Solid arrows* represent confirmed close contact with a known case during the period the case was considered infectious. *Broken arrows* present possible contact between the cases – they were in the same place at the same day but we cannot establish that they had close physical contact with each other. *Bi-directional arrows* indicate that we cannot accurately establish who was infected first. Time is presented as calendar weeks for the year 2020; week 12 beginning at 16 of March and week 24 ending at 14 of May. C, confirmed case; S, suspected case; pc, positive family contact; opc, positive contact outside home and work.

Figure 2 illustrates the time line of occurrence of symptoms for confirmed and suspected COVID-19 cases among HCWs. Of 50 cases, 27 (54%) reported that they had been at work during the time that they were, retrospectively, considered infectious. The median time from symptom onset to a positive test result was 3 days (IQR: 2–5). A transmission tree was developed based on contact tracing, showing possible links between COVID-19 cases and reported positive contacts (Fig. 3). Of 50 cases during the study period, 39 (78%) were directly connected to another co-worker at the ward (Fig. 3).

### Implementation of IPC measures

IPC measures at the ward gradually changed as the result of the pandemic and the outbreak of COVID-19 among HCWs. Based on information from the management and workplace meetings, IPC actions at different levels were compiled chronologically (Table 3).

**Table 3.** Infection control and prevention measures implemented at the ward

Level of IPC measures	IPC measures implemented	Date
Administrative level	Daily update meetings on COVID-19 and implementation of IPC measures with HCWs at the ward Daily update meetings on COVID-19 and IPC measures between the infectious ward management and managers from other departments at the hospital No visitors were allowed in the hospital Symptomatic HCWs were directed to be isolated at home for a minimum of 7 days The total number of HCWs at the ward was increased	From early March 2020 From early March 2020 11 March 2020 18 March 2020 28 March 2020
Means of physical distancing	Weekly social meeting – ‘Friday tea’ – at the ward was cancelled HCWs were urged to keep physical distance at all times, at least 1 m from one other Daily update meetings were conducted in two smaller groups instead of one big group Number of people in the staff lunch room was limited to 10 Sharing food and utensils in the lunch room were prohibited Specific signs were put up to indicate rooms with COVID-19 patients and the level of risk (e.g. if risk for aerosols)	7 April 2020 8 April 2020 9 April 2020 9 April 2020 14 April 2020 21 April 2020
Environment and personal hygiene routines	Increased frequency of disinfection of inanimate objects (computer keyboards, door handles, desk surfaces), together with active reinforcement of personal hygiene practices Increased availability of alcohol disinfections made it possible to place disinfections within reach at all workstations Extra resource from the cleaning department during weekends and evenings – cleaning of empty patient rooms Cleaning of empty patient rooms after treatment with high flow nasal cannula oxygen therapy could be done no sooner than 2 h after discontinuation of treatment (risk for aerosol) Shared stethoscopes in the patient's room were moved to the sluice room and disinfected after each use Personal hygiene routines emphasised at meetings – handwashing followed by disinfections before and after different working moments Personal hygiene routines emphasised at meetings – cleaning of surfaces both inside and outside patient rooms Extra resource from the cleaning department – increased frequencies of cleaning of shared areas such as lunch room, offices, corridors, kitchen area and toilets	24 March 2020 24 March 2020 27 March 2020 27 March 2020 27 March 2020 2–3 April 2020 7–9 April 2020 17–20 April 2020
PPE recommendations	PPE was recommended for any contact with the patient closer than 1.5 m Due to impending shortages, HCWs were required to share and re-use face shields after adequate disinfection Addition of surgical masks to be used together with face shields in close contact with infected patient and risk for splashes With more supplies arriving, the shared face shields were changed once daily, then eventually twice a day	19 March 2020 24 March 2020 3-6 April 2020 14-17 April 2020

IPC, infection prevention and control; PPE, personal protective equipment.

At the beginning of the study period, daily update meetings, comprising about 30 HCWs, were conducted in the staff dining room (~21 m^2^) (Supplementary Fig. S1). Shift change reports and patients' rounds, including three to five HCWs, were held in small offices (~10–12 m^2^). Cleaning and disinfection of rooms, computer keyboards and other inanimate objects was initially performed once daily by cleaning staff. At the beginning of the pandemic, HCWs were reminded daily to follow personal hygiene routines relating to droplet and contact transmission: washing hands and using alcohol disinfectant. PPE was used in contact with infected patients. Initially, full body coverage PPE was used as precaution. After the mode of COVID-19 transmission was established as predominantly droplet and contact (WHO 26 March 2020), HCWs were encouraged to disinfect hands before and after patient contact and to use a basic set of PPE (face shield, short-sleeved apron and single gloves). Aerosol generating procedures, such as tracheostomy care or high-flow nasal cannula oxygen therapy, required additional use of disposable particulate respirators class FFP2 or FFP3 (FFP: filtering face-piece).

### Knowledge of PPE guidelines

Self-reported knowledge of PPE use during four clinical scenarios was evaluated based on local recommendations at the time (Supplementary Fig. S3). A higher proportion of nurses and nurse aides scored 75% or 100% (70% and 73% respectively), compared to physicians (23%) and physiotherapists/rehabilitation assistants (60%). Ninety-six participants (69%) responded that they had at least once entered a patient room without the recommended PPE.

### HCWs' views on the outbreak

**Fig. 4.**
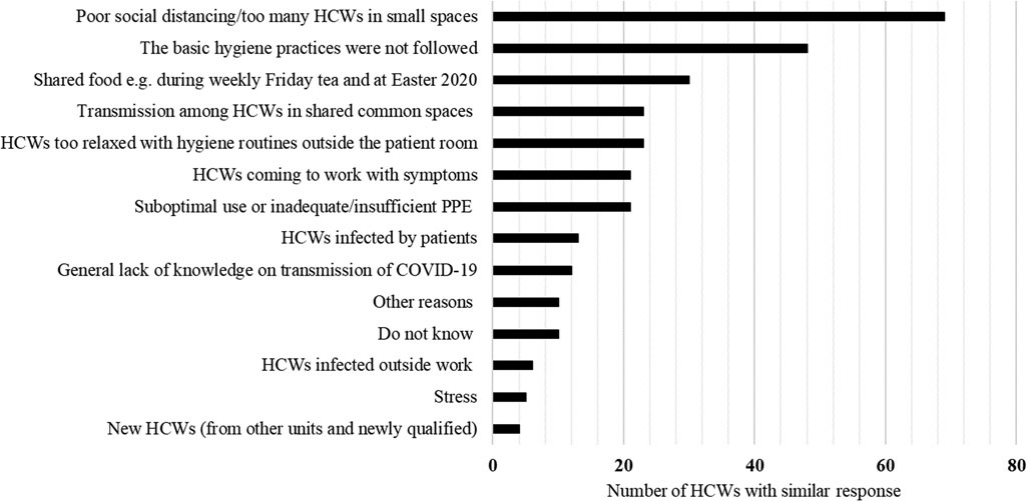
Reasons given by HCWs on why they thought there was an outbreak of COVID-19 among HCWs at the infectious disease ward. The graph summaries the answers in categories; more than one reason to the outbreak could be given. Altogether 131 of 152 HCWs answered to this open-ended question in the questionnaire.

In response to an open-ended question on why they thought there was a staff outbreak at the ward, HCWs reported lack of adequate physical distancing (*n* = 69; 45%) and not following the personal hygiene routines (*n* = 48; 32%) as the main reasons. Other reasons given included sharing food with work colleagues (*n* = 30; 20%) and HCWs not following hygiene routines outside the patient rooms (*n* = 23; 15%) (Fig. 4).

### The managers' views on IPC measures and the outbreak

During interviews with the managers, their views on IPC measures were explored (Supplementary Table S3). Local adaption and implementation of IPC measures were made in close collaboration between the Department of Infectious Diseases and the Department of Infection Control in the healthcare region. With increased knowledge on transmission routes (mid-end March), droplet and contact precautions were emphasised except for risk situations involving aerosol generation.

‘In the beginning, we used respirators, when we did not know if the virus was airborne, we always used long sleeves and gloves […]. Later we changed the PPE to the best protection against contact- and droplet transmission – face shields are the most important, and wearing an apron when you are close to the patient and gloves in contact with body fluids and otherwise short sleeves’ (Manager 2).

As part of IPC measures, one manager mentioned the intention to schedule enough numbers of HCWs to avoid a stressful working situation. Another manager concluded that nevertheless, increasing number of COVID-19 patients, the staff outbreak and the policy of home isolation if symptoms resulted in a shortage of staff at the ward.

Regarding means of transmission among HCWs', all three managers acknowledged increased risk of transmission in common spaces outside the patient rooms and the lack of physical distancing between HCWs.

‘The problem we recognize is how one [the staff] interacted when not being with the patients, that the staff might not have kept distance to each other and not been careful in preventing contact transmission’ (Manager 1).

One manager said that HCWs possibly came to work while experiencing mild symptoms, which could have contributed to the spread of COVID-19.

‘I think that we had persons with mild symptoms in the staff that we didn't know about… we somehow got the virus with us into areas outside the patient rooms and spread it between each other’(Manager 3).

Shortcomings in following IPC routines outside patient rooms were lifted, such as suboptimal cleaning and disinfection of surfaces and insufficient attention to personal hygiene practices. The practice of IPC measures was sometimes inadequate during transportation of patients within the hospital. In addition, the HCWs were initially less careful with the use of PPE during care of patients without confirmed COVID-19 infection, which led to unnecessary exposure. The managers described that the initial frequent changes in PPE recommendations were confusing and might have contributed to incorrect use of PPE. Shortages of face shields resulting in reuse several times might have contributed to transmission despite disinfection between each use.

One manager acknowledged that the management might have been late, and insufficient, in bringing awareness about the virus transmission amongst HCWs at the ward.

‘Concerning the transmission at the ward… I believe we might have informed less carefully about the spread of infection at the ward from the beginning. Even if we did, maybe we failed to emphasize the risk of transmission between staff’ (Manager 1).

Another manager reflected that there was initially a continuation of face to face work-related meetings in confined spaces at the ward. Retrospectively, these gatherings were not in line with the rapidly changing situation during the pandemic and may have increased the risk of transmission between HCWs.

‘We initially missed what was happening after we left the patient room, in our shared spaces, during our meetings…we didn't think about that. We gathered many people in crowded spaces and had updates and meetings’ (Manager 3).

The management maintained daily morning meetings with the local Department of Infection Control where strategies to manage the staff outbreak were discussed. The close cooperation with the local Department of Infection Control was mentioned as important for the management of the outbreak and the pandemic in general. It contributed to timely sharing of new information on COVID-19 and decisions on local recommendations.

Three main IPC strategies to stop the outbreak were identified in all three interviews: (1) reinforced physical distancing between HCWs, (2) enhanced focus on personal hygiene routines and (3) intensified cleaning of surfaces, in patient rooms and in shared areas used by HCWs. Reinforced physical distancing was implemented by separating HCWs during staff meetings, prohibiting too many people in common spaces and making HCWs aware of the risk of close contact. Enhanced focus on personal hygiene, primarily hand washing and hand disinfection, together with intensified cleaning of inanimate objectives. Handwashing and hand disinfection between work tasks was encouraged, also in areas outside patient rooms. One manager lifted that the outbreak put focus on the importance of staying at home also when having very discrete symptoms of infection. Cleaning was intensified by engaging the cleaning department in cleaning not only patient rooms, but also staff areas, as an attempt to prevent transmission between HCWs.

## Discussion

The context of this study was the first phase of the COVID-19 pandemic in Sweden in the spring of 2020. The knowledge of transmission routes of SARS-CoV-2 was limited and there was a global shortage of PPE [30]. A rapid transmission of SARS-CoV-2 among HCWs at an inpatient ward resulted in about one-third of the staff infected. This can be compared to the pooled prevalence of 11% among HCWs in a large meta-analysis from the first phase of the pandemic, however based on highly heterogeneous data sources [31]. In our study, contact tracing identified the HCWs themselves as a main source of the outbreak. Our findings emphasise HCW-to-HCW transmission in a hospital environment during the COVID-19 pandemic and support the measures of physical distancing, reinforcement of personal hygiene routines and surface cleaning to minimise infection transmission among HCWs.

The majority of HCWs being infected during the outbreak were categories spending the most time with infected patients, which has also been shown by others [17, 31, 32]. In our study, nurses and nurse aides, in addition to having the most frequent patient contact also spent the most time with each other. In the beginning of the study period, rounds, update meetings and meal breaks were held in rooms without space to practice physical distancing. PPE was only used in contact with patients with suspected or confirmed COVID-19 infection. This, together with the fact that some HCWs reported working whilst symptomatic, implies that they did not identify themselves as possible transmitters of COVID-19 at the time. Despite efforts from the management to recruit staff, there was a shortage of nurses and nurse aides during the study period and hard to find replacements. This possibly contributed to HCWs coming to work whilst symptomatic based on solidarity. In another study by our research group, HCWs at the ward expressed frustration of having to stay at home with only mild symptoms being aware of the high workload [33]. In addition to established knowledge of high viral load during the first symptomatic days, asymptomatic transmission was later acknowledged for SARS-CoV-2 [34, 35]. Contributing factors to transmission between HCWs were likely an unconscious relaxation in maintenance of hygiene routines outside the patient room as well as sub-optimal routines to reduce virus load on inanimate objects. Despite appropriate disinfection between uses, sharing of face shields at the beginning of the outbreak might have contributed to virus transmission. Reuse of PPE has been shown to increase the risk of COVID-19 infection among HCWs [10].

Based on the available data, it was impossible to conclude the primary source(s) of virus strain(s) transmitted among HCWs. Some HCWs reported unprotected contact with infectious, but undiagnosed, patients right at the beginning of the study period. However, we could not connect the major outbreak to these particular HCWs. Without genetic sequencing, it was also impossible to trace the virus strain(s) of the outbreak to patient exposure. To note, we are not aware of any HCW-to-patient transmission at the ward during the period. This suggests that within the patient rooms, IPC routines were followed adequately. We also found that HCWs working closer to infected patients, i.e. nurses and nurse aides, had more knowledge of PPE recommendations compared to other HCWs. At the time, the community prevalence of infection in Sweden was rising but based on limited test capacity the true magnitude of community and family exposure was impossible to establish. A study in a north American context (the Upper Midwest; USA), showed that based on genetic sequencing of 95 infected HCWs collected March to December 2020, community exposure was the predominant source of SARS-CoV-2 infections (57.9%), whereas only 4.2% of infections were traced to patients and 10.5% to co-workers [36]. Importantly, the cumulative community prevalence in the USA up to December 2020 was considerably higher than during the three first months of the pandemic in Sweden – also considering the low test capacity in Sweden at the time [37].

The main interventions to stop the COVID-19 outbreak at the ward were reinforcement of physical distancing between HCWs and emphasising personal hygiene routines, together with more rigorous cleaning of patient rooms and areas shared by HCWs. The importance of hand washing to protect against COVID-infection among HCWs was lifted early during the pandemic in a questionnaire-based study from a hospital setting in Wuhan [16]. Implementation of the mentioned interventions in our setting required behaviour changes among HCWs and demanded close and regular reminders and education, with special focus on introducing new staff to the current IPC routines. Working close with the local Department of Infection Control was important to identify and implement strategies to control the outbreak. The interventions required a minimum of economical and material resources. To note, the PPE recommendations were not changed in response to the staff outbreak and, by international comparison, a simplistic set of PPE was used at the time [38, 39]. By natural means, IPC recommendations and literature in general focus on protection of frontline HCWs from exposure to SARS-CoV-2 during patient contact. With studies indicating that up to 50% of virus exposure might occur in contact between HCWs, this transmission route is important to acknowledge [40, 41]. Preventing virus transmission between HCWs with the means of physical distancing, use of PPE between HCWs and cleaning shared areas outside patient rooms is seldom mentioned in the context of IPC management in a hospital environment [39]. These are simple and low-cost prevention measures, which are easy to replicate and especially beneficial in situations where there are resource constraints.

A strength of this study is the thorough attempt to map a COVID-19 outbreak among HCWs in a hospital environment by using multiple means of data collection. The fact that SARS-CoV-2 PCR testing was initially not available to HCWs most likely contributed to underestimation of the number of positive cases. The retrospective design using questionnaires can result in recall bias, something we aimed to compensate for by collecting data from multiple sources, e.g. laboratory test results. Another limitation was that the contact tracing was performed retrospectively and did not account for the duration of exposure to an infected colleague. By combining different data sources, e.g. the daily work schedule with individual case data on onset of symptoms and days worked with symptoms, we aimed to picture the transmission pathways as accurate as possible.

In summary, our data suggest that transmission of COVID-19 among HCWs during a major hospital outbreak predominantly occurred from HCW to HCW. The most important measures to prevent COVID-19 transmission among HCWs were enhanced awareness of the importance of physical distancing also outside the patient rooms and adherence to personal hygiene routines for droplet and contact transmission.

## Data Availability

The datasets used for the study are available from the corresponding author on reasonable request.

## Supplementary material

For supplementary material accompanying this paper visit https://doi.org/10.1017/S0950268822000231. click here to view supplementary material
