# Supplementary material for: Transmission of COVID-19 among healthcare workers-an epidemiological study during the first phase of the pandemic in Sweden
Source: Epidemiol Infect. 2022 Mar 11;150:e68. doi: 10.1017/S0950268822000231 (PMC8987659; doi:10.1017/S0950268822000231)
Supplement: Supplementary file 1 [file S0950268822000231sup001.docx]

**Journal:** Epidemiology and Infection

**Title:** Transmission of COVID-19 among healthcare workers - an epidemiological study during the first phase of the pandemic in Sweden

**Authors:** Sekai Chenai Mathabire Rücker, Catharina Gustavsson, Fredrik Rücker, Anders Lindblom, Maria Hårdstedt

**Supplementary material:**

Supplementary Figure S1

Supplementary Figure S2

Supplementary Figure S3

Supplementary Table S1

Supplementary Table S2

Supplementary Table S3

**Supplementary Figure S1.** Map of the ward at the Department of Infectious Diseases


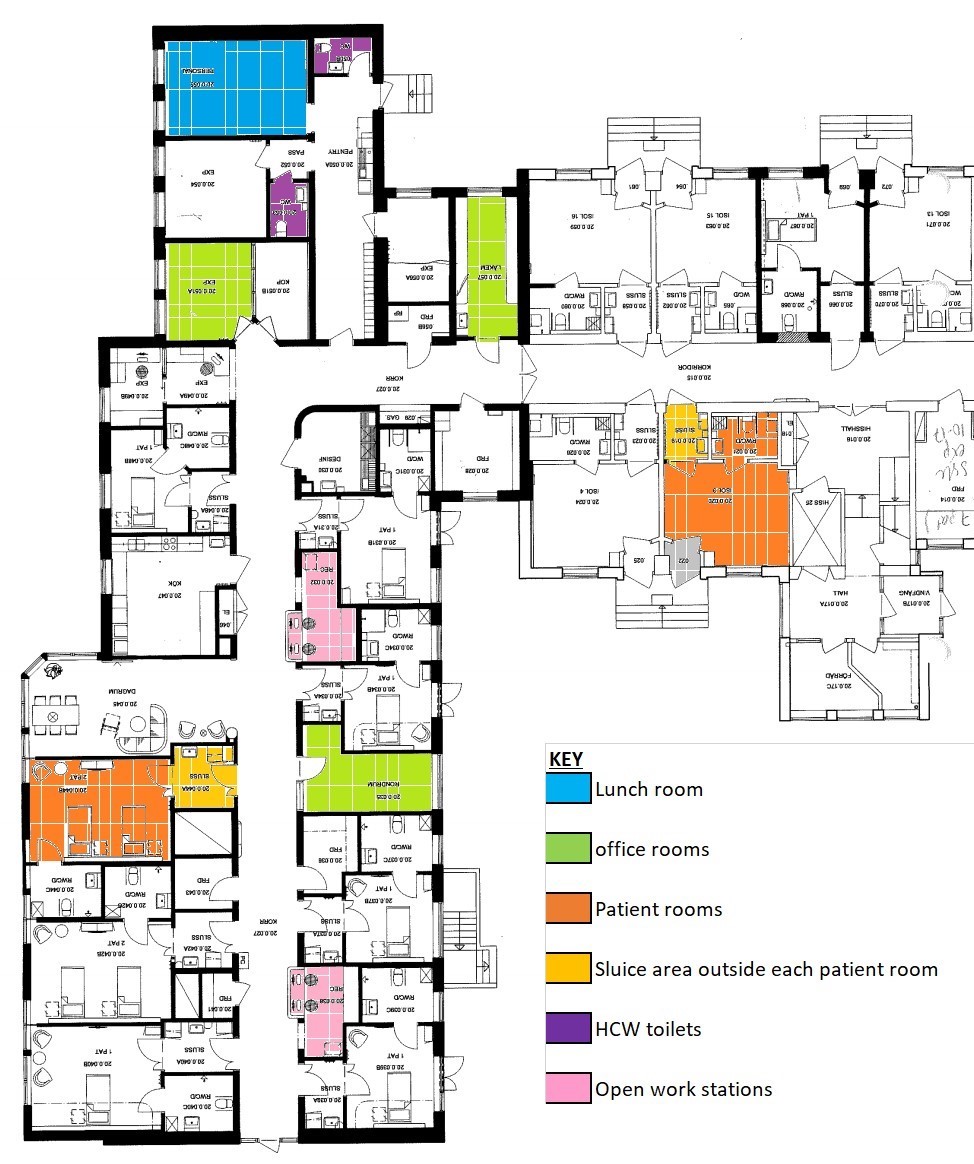


**Supplementary Figure S2**. Four hypothetical scenarios for evaluation of knowledge regarding correct PPE recommendations.

1. Please describe which combination of PPE that you have used in the following situations in the care of patients with COVID-19 at the ward
   1. Patient with COVID-19 receiving oxygen treatment – you are planning to go into the patient room and talk to the patient (at a distance of at least 1.5 meter):
      1. Disposable apron – short sleeve □ Yes □ No
      2. Disposable apron – long sleeve □ Yes □ No
      3. Plastic gloves □ Yes □ No
      4. FFP2/FFP3 respiratory mask □ Yes □ No
      5. Visor □ Yes □ No
      6. Mask □ Yes □ No
      7. No PPE is needed □ Yes □ No
      8. Other, specify_______________
      9. Do not know/Not assessable □
   2. Patient with COVID-19 receiving oxygen treatment – you are planning to go into the patient room to make the bed, performing lung auscultation, help with mobilisation of the patient, to clean the room or to take a venous blood sample:
      1. Disposable apron – short sleeve □ Yes □ No
      2. Disposable apron – long sleeve □ Yes □ No
      3. Plastic gloves □ Yes □ No
      4. FFP2/FFP3 respiratory mask □ Yes □ No
      5. Visor □ Yes □ No
      6. Mask □ Yes □ No
      7. No PPE is needed □ Yes □ No
      8. Other, specify_______________
      9. Do not know/Not assessable □
   3. Patient with COVID-19 and Optiflow/Airwo-treatment – you are planning to go into the patient room and talk to the patient (at a distance of at least 1.5 meter):
      1. Disposable apron – short sleeve □ Yes □ No
      2. Disposable apron – long sleeve □ Yes □ No
      3. Plastic gloves □ Yes □ No
      4. FFP2/FFP3 respiratory mask □ Yes □ No
      5. Visor □ Yes □ No
      6. Mask □ Yes □ No
      7. No PPE is needed □ Yes □ No
      8. Other, specify_______________
      9. Do not know/Not assessable □
   4. Patient with COVID-19 and Optiflow/Airwo-treatment – you are planning to go into the patient room to make the bed, performing lung auscultation, help with mobilisation of the patient, to clean the room or to take a venous blood sample:
      1. Disposable apron – short sleeve □ Yes □ No
      2. Disposable apron – long sleeve □ Yes □ No
      3. Plastic gloves □ Yes □ No
      4. FFP2/FFP3 respiratory mask □ Yes □ No
      5. Visor □ Yes □ No
      6. Mask □ Yes □ No
      7. No PPE is needed □ Yes □ No
      8. Other, specify_______________
      9. Do not know/Not assessable □

**Supplementary Figure S3:** Self-reported knowledge of personal protective equipment (PPE) use in four given theoretical scenarios (Supplementary Figure S2). Graphs presenting data for four different categories of HCWs: A) Physicians, B) Nurses, C) Nurse aides, and D) Physiotherapists/ Rehabilitation assistants.

**Supplementary Table 1: Detailed description of relevant contacts between HCWs as basis for contact tracing**

| **Categories** | **Short description** | **Detailed description** |
| --- | --- | --- |
| **A** | HCWs worked the same shift caring for different group of patients | HCWs working the same shift, caring for a different group of patients than the ‘infected colleague’. These HCWs met during tea and lunch breaks, they could respond to calls for help from other groups working on the same shift and they shared other common spaces e.g. corridor, toilet, patient kitchen, and other ward equipment. |
| **B** | HCWs worked the same shift caring for the same group of patients | HCWs working the same shift and caring for the same group of patients. These HCWs met several times in a small room to receive the patient report at (1) shift change, (2) after doctor’s round, (3) towards shift end to evaluate the day’s work. During the study period, no masks were used during these sessions. |
| **C** | HCWs cared for the same group of patients in consecutive shifts (meeting for handover reports) | HCWs caring for the same group of patients in consecutive shifts: the nurse ending the shift would meet all the staff working the next shift in a closed room to give the handover report. There was also an overlap of one-two hours were these HCWs worked together, also possibility sitting together in the common areas to have tea. |

**Supplementary Table S2:** Background characteristics of study participants

| **Characteristic** | **Physicians (n=22)** | **Nurses**  **(n=57)** | **Nurse aides (n=41)** | **Cleaning staff (n=21)** | **Physiotherapists/**  **Rehabilitation assistants (n=5)** | **Medical secretaries (n=4)** | **Kitchen assistants**  **(n=2)** | **Total**  **(n=152)** |
| --- | --- | --- | --- | --- | --- | --- | --- | --- |
| **Female**; n (%) | 9 (41) | 56 (98) | 38 (93) | 19 (90) | 3 (60) | 4 (100) | 1 (50) | 130 (86) |
| **Age**: median (IQR) | 38 (34-51) | 33 (28-39)^d^ | 37 (26-55) | 26 (22-28) | 53 (38-53) | 51 (37-62) | 19 (18-19) | 33 (26-47)^d^ |
| **Work experience, years**: median (IQR) | 10 (3-23) | 7 (3-10)^b^ | 7 (2-31)^a^ | 3 (0-6)^d^ | 20 (10-27) | 19 (10-30) | 1 (1-1) | 7 (2-18)^g^ |
| **Worked entire study period**; n (%) | 16 (77) | 24 (42) | 22 (54) | 10 (48) | 1 (20) | 2 (50) | 0 | 83 (55) |
| **Days worked if not during the whole study period**:  Mean (SD) | 48 (32) | 40 (38) | 35 (26) | 47 (12) | 41 (3) | 41 (3) | 19 (6) | 40 (31) |
| **Regular place of work** |  |  |  |  |  |  |  |  |
| Infectious disease ward; n (%) | 15 (68) | 29 (51) | 23 (56) | 13 (62) | 1 (20) | 0 | 2 (100) | 83 (55) |
| Other clinical unit^ⱡ^; n (%) | 4 (18) | 24 (42) | 10 (24) | 1 (5) | 3 (60) | 4 (100) | 0 | 46 (30) |
| Other non-clinical^¥^; n (%) | 3 (14) | 4 (7) | 8 (20) | 7 (33) | 1 (20) | 0 | 0 | 23 (15) |

Missing values: a=1, b=2, c=3, d=4, g=7

ⱡOther clinical unit includes other hospital inpatient and outpatient units, as well as primary care units

¥ Other non- clinical unit includes working at the laboratory, infection control department, retired and unemployed

**Supplementary Table S3: Categories and subcategories identified by content analysis of interviews with the managers**

| **Main categories** | **Subcategories** |
| --- | --- |
| Rationales for IPC routines | Based on recommendations from The Public Health Agency of Sweden and the National Board of Health  Local adaption and implementation in dialogue between Dept. Infectious Disease and Dept. of Infection Control  Focus on droplet and contact transmission |
| Means of transmission during the infectious outbreak among HCWs | Transmission outside the patient rooms  HCWs working with symptoms  Shortcomings in following IPC routines at the ward  Reuse of face shields due to shortage  Shortcoming by the management - delayed information about the outbreak to the staff and implementation of changes in IPC measures |
| IPC strategies applied to stop the outbreak | Reinforced physical distancing between HCWs at the ward  Enhanced focus on personal hygiene routines  Intensified cleaning of common surfaces at the ward |
